# A nationwide analysis of the treatment patterns, survival, and medical costs in Korean patients with relapsed or refractory diffuse large B-cell lymphoma

**DOI:** 10.3389/fonc.2024.1282323

**Published:** 2024-02-01

**Authors:** Jeong-Yeon Cho, Suk-Chan Jang, Dong-Won Kang, Eui-Kyung Lee, Hyein Koh, Dok Hyun Yoon, Mi-Hai Park

**Affiliations:** ^1^ School of Pharmacy, Sungkyunkwan University, Suwon, Gyeonggi-do, Republic of Korea; ^2^ Novartis, Seoul, Republic of Korea; ^3^ Department of Oncology, Asan Medical Center, University of Ulsan College of Medicine, Seoul, Republic of Korea

**Keywords:** diffuse large B-cell lymphoma, hematopoietic stem cell transplantation, treatment patterns, survival, medical costs

## Abstract

**Background:**

Approximately one-third of patients with diffuse large B-cell lymphoma (DLBCL) are refractory to treatment or experience relapse after initial therapy. Unfortunately, treatment options for older patients and those who experience relapse or become refractory to hematopoietic stem cell transplantation (HSCT) are limited. This nationwide population-based study aimed to identify treatment patterns, survival times, and treatment costs in patients with relapsed/refractory DLBCL (R/R DLBCL).

**Materials and methods:**

Between 2011 and 2020, data on patients with R/R DLBCL were retrieved from the Korean Health Insurance Review & Assessment Service, encompassing the entire population. We identified the treatment patterns for each treatment line using a Sankey diagram and calculated the median time to the subsequent treatment in line. Median overall and progression-free survival times were estimated using the Kaplan–Meier survival curves. Finally, the medical costs incurred during DLBCL treatment were calculated for each treatment line and the costs related to HSCT were summarized at the episode level.

**Results:**

A total of 864 patients with R/R DLBCL who received second-line treatment were identified, and a regimen of ifosfamide, carboplatin, and etoposide (ICE) was administered the most. Among them, 353 were refractory or relapsed cases that were treated with third-line treatments. The median times for second-line to third-line, third-line to fourth-line, fourth-line to fifth-line, and fifth-line to sixth-line treatment failures gradually decreased (3.93, 2.86, 1.81, and 1.38 months, respectively). The median overall survival time was 8.90 and 4.73 months following the second-line and third-line treatments, respectively. In the third-line treatment setting, the patients did not show a significant difference in survival time after HSCT. The median medical cost was $39,491 across all treatment lines including the cost of HSCT which was $22,054.

**Conclusion:**

The treatment patterns in patients with R/R DLBCL, especially at third-line treatments and thereafter, were complicated, and their prognosis was poor despite the high medical costs. Novel and effective treatment options are expected to improve the prognosis and alleviate the economic burden of patients with R/R DLBCL.

## 1 Introduction

Diffuse large B-cell lymphoma (DLBCL) accounts for approximately 30% of non-Hodgkin lymphoma (NHL) cases, with an age-adjusted incidence of 5.0 cases per 100,000 person-years worldwide (1, 2). Although DLBCL affects patients of all ages, it is most common in patients aged > 60 years (3). The rituximab, cyclophosphamide, hydroxydaunorubicin, oncovin, and prednisone (R-CHOP) regimen was introduced as a standard first-line treatment in 2002, and a polatuzumab-vedotin combination regimen with rituximab, cyclophosphamide, hydroxydaunorubicin, and prednisone (R-CHP) was recently introduced as a first-line treatment (4, 5). However, approximately one-third of patients experience relapse or disease progression after first-line treatment, and 83% of progression occurs within the first 3 years of treatment (6). For patients with relapsed/refractory DLBCL (R/R DLBCL), high-dose therapy with autologous hematopoietic stem cell transplantation (HDT/HSCT) is recommended (7, 8). However, no clear treatment options are available for patients ineligible for HSCT because of older age, frailty, lack of response to second-line treatment, or failure to collect stem cells (9). Furthermore, the treatment strategy is less apparent in these patients, particularly after the failure of a second-line treatment (10). Although not yet widely available, the introduction of bispecific antibody therapies and chimeric antigen receptor (CAR) T-cell therapies for patients with R/R DLBCL is anticipated to expand treatment options, potentially improving prognoses and alleviating the economic burden on these patients (11–15).

A previous study in the United States reported that rituximab-based regimens were the most prevalent, with 25% of patients receiving HSCT as a second-line treatment (16). Another study demonstrated that the median survival of patients with R/R DLBCL was 13.4 months after the initiation of second-line treatment in an outpatient setting (17). Nevertheless, only a few studies have reported on the survival times and treatment patterns of patients with R/R DLBCL, particularly those who have received second- to third-line treatments in real-world settings. In addition, studies using nationwide Korean data on these patients are limited, and treatment patterns differ from country to country depending on the reimbursement system. Therefore, we aimed to identify the treatment patterns and survival of patients with R/R DLBCL and analyze the economic burden using Korean claims data from the Health Insurance Review and Assessment Service (HIRA).

## 2 Materials and methods

### 2.1 Study design and data source

We performed a retrospective observational study using the HIRA claims data, which contain data on more than 98% of the nationwide population in South Korea (18). The data included patient characteristics such as age, sex, prescribed medications, medical procedures reimbursed by the National Health Insurance Service (NHIS), and disease codes according to the Korean Classification of Disease 7th version (KCD-7), which is a modified version of the International Classification of Disease 10th version (ICD-10). In Korean claims data, the overall positive predictive value of diagnosis using ICD-10 codes is 82% (19). Data from January 1, 2011, to February 28, 2020, were analyzed in our study, and the enrollment period during which patients with DLBCL were identified was from January 1, 2013, to December 31, 2019.

### 2.2 Study population and eligibility criteria

The target population for this study was patients with R/R DLBCL, defined as those who received second-line treatments. Patients who died without receiving second-line treatments were excluded. Prior to the selection of patients with R/R DLBCL, we constructed a cohort of patients who were newly diagnosed with DLBCL during the index period from January 1, 2013, to December 31, 2019, using their diagnosis codes and medical history claims for diffuse large B-cell lymphoma (DLBCL) (ICD-10 codes C83.3). The index date was defined as the first record of a newly diagnosed DLBCL. To exclude confounding diseases and overcome the limitations of our data, we excluded patients who met the following criteria: (1) patients who had a history of DLBCL within 5 years before the index date (washout period); (2) patients who had a history of confounding lymphomas, such as small cell B-cell lymphoma (C83.0), mantle cell lymphoma (C83.1), lymphoblastic lymphoma (C83.5), Burkitt lymphoma (C83.7), other non-follicular lymphomas (C83.8), primary mediastinum large B-cell lymphoma (C85.2), and solid cancer (C00–C80) during the study period; (3) patients with confounding medical histories, such as a history of HSCT before the index date; (4) patients with no treatment records after the index date; (5) patients who had a record of DLBCL within 2 years before the index date or who had a record of salvage chemotherapy regimens as the first-line treatment. This last criterion was created to account for patients with washout periods of less than five years prior to study inclusion.

To select eligible patients with R/R DLBCL, the treatment regimen was defined by combining the drugs administered for each medical episode. The specific medications and regimens used are summarized in **Table S1**. Moreover, we determined whether the treatment regimen for each medical episode was the same as that of the previous regimen based on the combination of drugs. Treatments targeting central nervous system diseases, such as the administration of intrathecal methotrexate or modifications in corticosteroid prescriptions, were considered independent of the line of treatment. We then used both treatment regimens and gaps between treatment episodes to classify the treatment line. First-line treatment was defined as prescribed medications for 12 weeks from the first record of newly diagnosed DLBCL (17). Second-line treatment was defined as the first record of switching the treatment regimen from first-line treatment. Each treatment line was defined similarly. However, if patients received the HDT regimen before HSCT, the HDT and HSCT were considered consecutive within the same treatment line (HDT/HSCT). Follow-up began from the date of each line of treatment and continued until death or the end of the study (February 28, 2020), during which time only patients with claims data were selected. All patients were followed-up for at least 60 days.

### 2.3 Outcomes and measurement

Baseline characteristics included age, sex, and comorbidities within 1 year before the index date. Age groups were stratified based on the eligibility for HSCT according to the local reimbursement criteria, which were up to 65 years of age during the study period. We assessed treatment patterns using the medical records of each patient to identify their treatment lines and survival data.

We calculated the median time to the next treatment (TTNT) for each treatment line and estimated the patients’ overall survival (OS), which was defined as the time from initiating each line of treatment until death. The claims data of the HIRA contained only “in-hospital” deaths. Therefore, if mortality was only marked by the “in-hospital death” code, patient survival rates would be highly overestimated. To address this limitation, we defined “out-of-hospital” death as the date of the last claims filed for patients with no further records for 6 months, a method that has been adopted in previous research and validated in high-mortality cancer patients (20, 21). We then assessed the survival outcomes from the first date of each line of treatment. In addition to OS, progression-free survival was defined as the survival time from the date of relapse or refractory disease diagnosis to the initiation of a subsequent line of treatment or death.

Medical costs incurred during DLBCL treatment were summarized for each treatment line. In contrast, the costs related to HSCT were summarized at the episode level to determine the total economic burden. We also calculated cumulative medical costs while considering censoring (22) to show the difference in disease-related costs between patients who received third-line treatments and those who did not. Costs in South Korean Won (KRW) were converted to United States Dollar (USD) at the 2020 exchange rate of 1,086.3 KRW/USD.

### 2.4 Statistical analysis

Descriptive analyses were performed to assess patient demographics, survival, treatment patterns, and medical costs. Categorical variables were expressed as counts and percentages of patients in each category, whereas continuous variables were expressed as mean and standard deviation (SD) or median and interquartile range (IQR). The Charlson Comorbidity Index (CCI) was estimated to include the risk for the severity of the underlying disease before diagnosis (23, 24). Survival analysis was performed to estimate the survival probability over time and calculate the survival time. The median survival time of patients with R/R DLBCL was computed using the Kaplan–Meier curve with a 95% confidence interval (CI). All analyses were performed using SAS version 9.4 (SAS Institute, Cary, NC, USA).

## 3 Results

### 3.1 Baseline characteristics of patients with R/R DLBCL

**Figure 1:**
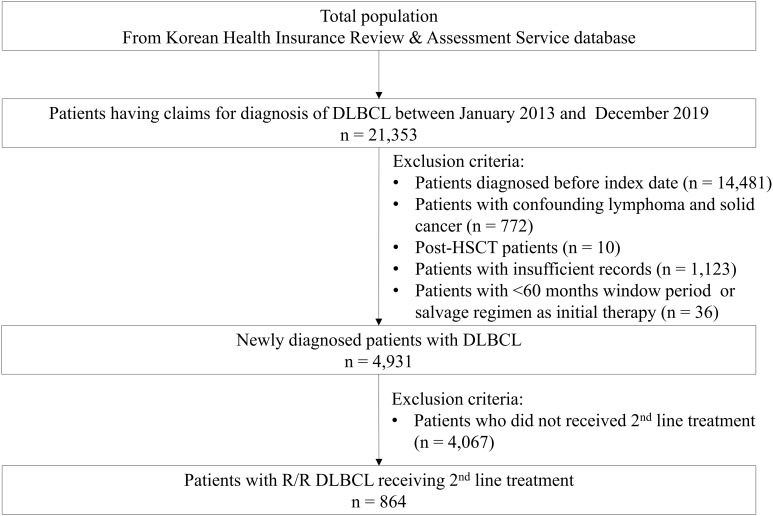
Flow chart of the selection process for eligible patients with R/R DLBCL.

A total of 21,353 patients were diagnosed with DLBCL between January 2013 and December 2019, and 4,931 eligible patients with newly diagnosed DLBCL were identified. Among them, 4,067 patients were excluded because they did not receive second-line treatment including 922 patients who died of progressive disease or other causes. Finally, 864 patients who experienced relapsed or refractory DLBCL and received second-line treatments were selected for this study (**Figure 1**). The baseline characteristics of the patients included in this study are presented in **Table 1**. The median (IQR) age of the population was 63 (53–71) years; more than half of the patients met the age criteria for HSCT (55.79%), and the male patients (n = 522, 60.42%) outnumbered the female patients.

**Table 1:** Baseline characteristics of patients with R/R DLBCL.

Variables	Second-line treatment (N = 864)	Third-line treatment (N = 353)
Age, median (IQR)	63 (53–71)	60 (50–68)
Age group, n (%) ^a)^		
< 65	482 (55.79)	222 (62.89)
≥ 65	382 (44.21)	131 (37.11)
Sex, n (%)		
Male	522 (60.42)	223 (63.17)
Female	342 (39.58)	130 (36.83)
CCI, median (IQR)	5.00 (4.00–8.00)	5.00 (4.00–8.00)
Comorbidities, n (%)		
Diabetes	368 (42.59)	150 (42.49)
Hypertension	405 (46.88)	155 (43.91)
Heart disease ^b)^	74 (8.56)	32 (9.07)

^a)^Age eligibility for HSCT according to the local reimbursement criterion: < 65 years.

^b)^Heart disease was defined by ICD-10 codes I21 (acute MI), I22 (STEMI), I43 (cardiomyopathy), and I50 (HF).

HSCT, hematopoietic stem cell transplantation; R/R DLBCL, relapsed or refractory diffuse large B-cell lymphoma; CCI, Charlson comorbidity index.

### 3.2 Treatment patterns of R/R DLBCL

**Figure 2:**
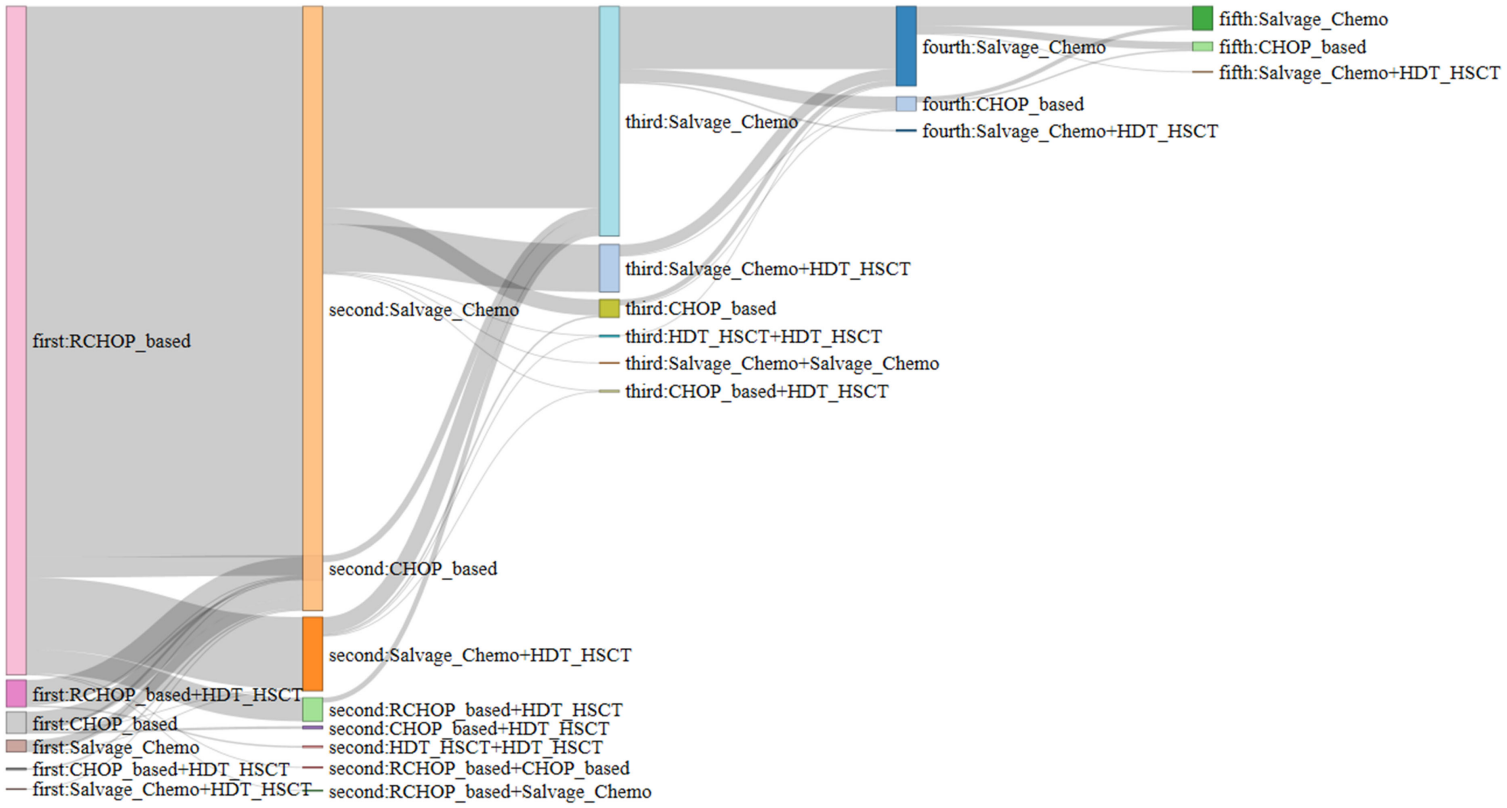
Treatment patterns for patients with R/R DLBCL.

**Figure 2** displays the treatment patterns of the patients with R/R DLBCL. Of the 864 patients, 821 (95.02%) received R-CHOP-based regimens as the first-line treatment and 32 underwent HDT/HSCT as a consolidation therapy (**Table S1**). In terms of second-line treatments, 363 (42.01%) patients received an ifosfamide, carboplatin, and etoposide (ICE) regimen, of whom 42 additionally underwent HDT/HSCT. Other second-line treatments included the etoposide, methylprednisolone, cytarabine, and cisplatin (ESHAP) (16.09%) and dexamethasone, cytarabine, and cisplatin (DHAP) regimens (15.39%). Regarding third-line treatments, 82 patients (23.23%) received the DHAP regimen, 21.81% received ICE, and 14.16% received the mesna, ifosfamide, mitoxantrone, and etoposide (MINE) regimen. During the follow-up period, 212 patients (24.54%) underwent HSCT.

### 3.3 Time to next treatments and survival of R/R DLBCL patients

**Figure 3:**
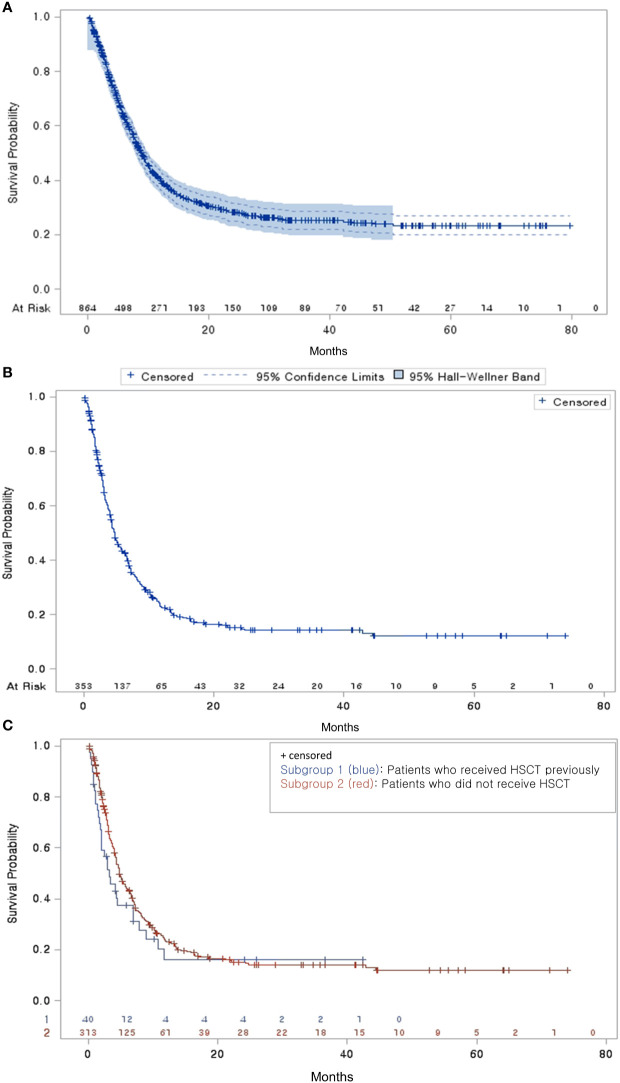
Overall survival probability **(A)** Overall survival of patients with R/R DLBCL who received second-line treatment **(B)** Overall survival of third-line treatment patients from the end of their second-line treatment **(C)** Overall survival of third-line treatment patients by HSCT subgroup.

The median time from diagnosis to a second-line treatment was 9.33 months (**Table 2**). Among the 864 patients with R/R DBLCL, 353 experienced progression after second-line treatments and received third-line treatments. The median time from a second-line to a third-line treatment was 3.93 months. Among them, 330 (93.48%) received salvage chemotherapy (58 patients with HDT/HSCT and 272 without HDT/HSCT). A total of 114 and 42 patients experienced third- and fourth-line treatment failures, respectively. The median times for third-line to fourth-line, fourth-line to fifth-line, and fifth-line to sixth-line treatment failures gradually decreased (2.86, 1.81, and 1.38 months, respectively). Most patients received salvage chemotherapy in each line of treatment (93.48%, 85.09%, and 73.81%, respectively). The median OS time was 8.90 months after a second-line treatment and 4.73 months after a third-line treatment (**Figures 3A, B**). Regardless of the previous administration of HSCT, third-line treatments did not significantly differ in terms of median OS times (3.31 vs. 4.83 months, p = 0.242) (**Figure 3C**).

**Table 2:** Time to next treatments and survival of R/R DLBCL patients.

	Patients with R/R DLBCL
**Median follow-up time, months (IQR)**	*7.62 (3.55–15.33)*
Median time to next treatment, months (IQR)	
Time from diagnosis to second-line treatment (n = 864)	9.33 (5.98–16.46)
Time from second-line treatment to third-line treatment (n = 864)	3.93 (2.00–9.89)
Time from third-line treatment to fourth-line treatment (n = 353)	2.86 (1.38–6.64)
Time from fourth-line treatment to fifth-line treatment (n= 114)	1.81 (1.08–3.19)
Time from fifth-line treatment to sixth-line treatment (n = 42)	1.38 (0.95–4.34)
Overall survival	
Median time from second-line treatment, months (95% CI), (n = 864)	*8.90 (8.08, 9.63)*
% proportion of censored patients	*34.38*
Median time from third-line treatment, months (95% CI), (n = 353)	4.73 (4.17, 5.72)
% proportion of censored patients	21.81
Progression-free survival	
Median time from second-line treatment, months (95% CI), (n = 864)	*4.47 (3.88, 5.06)*
% proportion of censored patients	*25.46*
% proportion of death before progression	*33.68*
Median time from third-line treatment, months (95% CI), (n = 353)	3.12 (2.73, 3.61)
% proportion of censored patients	17.85
% proportion of death before progression	49.86
Number of patients who received HSCT, n (%) (n = 864)	212 (24.54)
Autologous HSCT	210 (24.31)
Allogenic HSCT	9 (1.04)

HSCT, hematopoietic stem cell transplantation; R/R DLBCL, relapsed or refractory diffuse large B-cell lymphoma.

### 3.4 Economic burden of R/R DLBCL patients

The total lifetime medical cost across all treatment lines for R/R DLBCL was $39,491, and the cost related to HSCT was $22,054 (**Table 3**). The cost for patients who experienced second-line failures was $42,706, whereas it was $34,182 for those who did not encounter second-line failure (**Table S2**). The median cost of each treatment line was the highest from diagnosis to second-line treatment ($32,468). Among subsequent treatment lines, the cost from second-line to third-line treatment was the highest ($21,058), followed by costs from third-, fourth-, and fifth-line treatments to subsequent-line treatments. After the failure of second-line treatments, the cumulative cost for patients who received third-line treatments was higher than that of patients who did not receive third-line treatment from the fifth month onwards (**Figure S1** and **Table S3**).

**Table 3:** Economic burden of patients with R/R DLBCL.

	Patients with R/R DLBCL
**Total medical cost, median (IQR) (n = 864)**	$39,491 (21,451–62,732)
Total inpatient cost	$31,069 (15,791–52,685)
Total medical cost related to HSCT	$22,054 (15,804–32,040)
Medical costs for each treatment line, median (IQR)	
From diagnosis to second-line treatment (n = 864)	$32,468 (26,174–41,667)
From second-line treatment to third-line treatment (n = 864)	$21,058 (11,456–38,154)
From third-line treatment to fourth-line treatment (n = 353)	$21,017 (10,197–39,718)
From fourth-line treatment to fifth-line treatment (n= 114)	$18,228 (8,658–35,140)
From fifth-line treatment to sixth-line treatment (n = 42)	$16,147 (2,778–32,631)

R/R DLBCL, relapsed or refractory diffuse large B-cell lymphoma; IQR, interquartile range; 1 USD = 1086.30 KRW (2020 exchange rate).

## 4 Discussion

In this nationwide population-based study, we estimated the treatment patterns, survival times, and treatment costs for patients with R/R DLBCL. Our findings revealed a lack of clear treatment patterns for these patients, with the ICE, ESHAP, and DHAP regimens being the most commonly administered, in that specific order. The median OS of patients with R/R DLBCL was 8.90 months and the progression-free survival time was 4.47 months. Additionally, we determined that the median treatment cost for these patients was $39,491 over their lifetime. As the patients experienced multiple treatment failures and received subsequent lines of therapy, a decreasing trend in TTNT was observed. Although TTNTs decreased remarkably in subsequent treatment stages, the decrease in cost was relatively minor.

The treatment of R/R DLBCL remains a clinical challenge. R-CHOP has been the standard first-line treatment for DLBCL for more than 20 years (25, 26), and a polatuzumab-vedotin combination regimen has recently been introduced (4). However, an established effective treatment for patients with R/R DLBCL, particularly those who cannot undergo transplantation, remains lacking (27). Similar to a previous study (28), approximately one-fourth of the patients in this study received HSCT, and many patients only underwent salvage chemotherapy due to the lack of treatment options for DLBCL within the reimbursement criteria in Korea.

Although several studies have analyzed the survival of patients with DLBCL, few have investigated patients experiencing relapsed or refractory disease. The median OS of 6.3 months presented by Crump et al. (29) was similar to that in our study, considering that they focused on patients who received only salvage chemotherapy. However, this study reported only OS after a second-line treatment, whereas our study holds significance for presenting OS following a third-line treatment in patients with second-line failure. Another study investigated outpatient chemotherapy in patients with R/R DLBCL; however, our study adds value by including both inpatients and outpatients (17). We also presented OS with a focus on HDT combined with HSCT, which is recommended as a second-line treatment for patients with chemotherapy-sensitive DLBCL (7). Patients with R/R DLBCL who underwent HSCT exhibited a better prognosis than those who did not (30). However, because the prognosis for relapse after HSCT is poor, similar to other second-line options, HSCT should be carefully considered.

The median TTNTs rapidly shortened after the first relapse or refractory diagnosis and continued to decrease until the sixth-line treatment. In other words, as the disease progressed, the response to the drug decreased, resulting in a rapid occurrence of refractoriness or relapse. Although the TTNTs continued to decrease until the sixth-line treatment, the medical costs were similar, indicating that the cost per unit time was higher on the next subsequent treatment line than on the prior treatments and that the economic burden increased as the treatment failed. This was presumably because of the absence of other anticancer therapy and the availability of only salvage chemotherapy; therefore, the medical expenses required to provide care for patients eventually increased. A previous study reported that patients who experienced relapse spent significantly more on medical costs than those who did not experience relapse (31). From the perspective of each treatment line, the higher cost of each treatment line compared with that of the next treatment line could be attributed to the TTNT. A previous study demonstrated that the cost of the treatment for relapse after 3 months was higher than that of relapse within 3 months (31). Although several studies have estimated the medical costs in patients with DLBCL, most of them only demonstrated the medical costs for treating DLBCL and not for R/R DLBCL. In a study evaluating the medical costs in patients with R/R DLBCL, a similarly high cost was observed (31). However, their assessment was confined to the initial few years post-diagnosis; therefore, we supplemented this by calculating the total lifetime costs. Patients who experienced relapse after the second-line treatment spent more disease-related costs across all time points than that patients who did not experience relapse.

This study had several limitations. Although the study lacked clinical information, such as patients’ disease stages and treatment lines, we classified their treatment regimens and lines based on the drugs in each of their claims and the intervals between claims. Therefore, it is possible that some treatment lines were misclassified, which may have affected the TTNT and medical expenses. In addition, patients with R/R DLBCL who did not receive second-line treatment because of relapse or refractory disease may have been excluded. Third, the claims data did not allow us to identify non-covered drugs. Therefore, treatment costs may have been underestimated. Another limitation of this study was that we could not include newly introduced therapies because of the limited study period. For instance, starting in April 2022 in South Korea, CAR T-cell therapies were reimbursed for patients who experienced failure with second-line treatment and those who faced failure after HSCT (32). Since these therapies have shown clinical benefits through trials to improve the prognosis of patients with R/R DLBCL (11–13), they are likely to affect treatment patterns after reimbursement (33). However, CAR T-cell therapies could not be included because they were introduced after the study period (34). With the recent approval of other CAR T-cell and bispecific antibody therapies (14, 15), the treatment paradigm for patients with R/R DLBCL is expected to change in the future, and further long-term follow-up studies, including novel therapies, should be conducted after data accumulation. Therefore, although our data did not include newly introduced therapies, our results remain valuable because they offer insights into the population to which new treatments will be applied.

Despite these limitations, our study had significant strengths. We assessed the clinical and economic burden of patients with R/R DLBCL using long-term real-world data derived from the nationwide HIRA database encompassing the entire national population. In addition, this study adequately evaluated the disease burden of patients by focusing on those with R/R DLBCL. Unlike previous studies that reported only treatment regimen ratios (16, 17, 35), our study demonstrated the flow of regimens using a Sankey diagram and reported their complexities.

## 5 Conclusion

Complex treatment patterns, poor prognoses, and high medical costs have been reaffirmed by the results of previous studies on patients with R/R DLBCL, especially those who received third-line treatments. This high clinical and economic burden in patients with R/R DLBCL may be due to limited treatment options following second-line treatments. Establishing appropriate policies and novel treatment options that will provide excellent response rates is expected to improve prognosis and alleviate the economic burden of patients with R/R DLBCL.

## Data availability statement

Publicly available datasets were analyzed in this study. This data can be found here: Data from the Korean National Health Insurance Service (M20200325409) were obtained after appropriate authorization approval. (https://opendata.hira.or.kr/).

## Ethics statement

The Institutional Review Board of Sungkyunkwan University approved this retrospective study (SKKU-2020-01-010). Written informed consent from the participant’s legal guardian/next of kin was not required to participate in this study in accordance with the national legislation and institutional requirements. The studies were conducted in accordance with the local legislation and institutional requirements. The ethics committee/institutional review board waived the requirement of written informed consent for participation from the participants or the participants’ legal guardians/next of kin because As participants’ personal information was anonymized, the institutional review board waived the need for informed consent for this study.

## Author contributions

J-YC: Conceptualization, Data curation, Formal analysis, Investigation, Methodology, Writing – original draft. S-CJ: Conceptualization, Formal analysis, Investigation, Writing – original draft, Writing – review & editing. D-WK: Investigation, Writing – original draft. E-KL: Supervision, Writing – review & editing. HK: Conceptualization, Writing – review & editing. DHY: Conceptualization, Validation, Writing – review & editing. M-HP: Conceptualization, Funding acquisition, Project administration, Supervision, Writing – review & editing.
