# Supplementary material for: A nationwide analysis of the treatment patterns, survival, and medical costs in Korean patients with relapsed or refractory diffuse large B-cell lymphoma
Source: Front Oncol. 2024 Feb 1;14:1282323. doi: 10.3389/fonc.2024.1282323 (PMC10867264; doi:10.3389/fonc.2024.1282323)
Supplement: Supplementary file 1 [file DataSheet_1.docx]

Supplementary Material

**A nationwide analysis of the treatment patterns, survival, and medical costs in Korean patients with relapsed or refractory diffuse large B-cell lymphoma**

**Jeong-Yeon Cho^1†^, Suk-Chan Jang^1†^, Dong-Won Kang^1^, Eui-Kyung Lee^1^, Hyein Koh^2^, Dok Hyun Yoon^3*^, and Mi-Hai Park^1*^**

†These authors have contributed equally to this work and share first authorship.

^1^School of Pharmacy, Sungkyunkwan University, Suwon, Gyeonggi-do, Republic of Korea

^2^Novartis, Seoul, Republic of Korea

^3^Department of Oncology, Asan Medical Center, University of Ulsan College of Medicine, Seoul, Republic of Korea

*** Correspondence:**Mi-Hai Park, PhD
[bestway00@skku.edu](mailto:bestway00@skku.edu)

Dok Hyun Yoon, MD, PhD
[dhyoon@amc.seoul.kr](mailto:dhyoon@amc.seoul.kr)

Supplementary Table S1. Proportion of treatment regimens by the line of treatment

| **Treatment regimen** | **First-line**  **(N = 864)** | **Second-line**  **(N = 864)** | **Third-line**  **(N = 353)** | **Fourth-line**  **(N = 114)** | **Fifth-line**  **(N = 42)** |
| --- | --- | --- | --- | --- | --- |
| R-CHOP-like ^a)^ | 789 (91.3) | 1 (0.1) | 0 (0.0) | 0 (0.0) | 0 (0.0) |
| R-CHOP-like followed by HSCT | 32 (3.7) | 28 (3.2) | 0 (0.0) | 0 (0.0) | 0 (0.0) |
| CHOP-like ^a)^ | 26 (3.0) | 29 (3.4) | 21 (6.0) | 17 (15.0) | 11 (26.8) |
| CHOP-like followed by HSCT | 2 (0.2) | 3 (0.4) | 2 (0.6) | 0 (0.0) | 0 (0.0) |
| ICE | 2 (0.2) | 321 (37.1) | 61 (17.3) | 13 (11.5) | 2 (4.9) |
| ICE followed by HSCT | 0 (0.0) | 42 (4.9) | 16 (4.5) | 0 (0.0) | 0 (0.0) |
| DHAP | 1 (0.1) | 113 (13.1) | 72 (20.4) | 16 (14.2) | 1 (2.4) |
| DHAP followed by HSCT | 0 (0.0) | 20 (2.3) | 10 (2.8) | 1 (0.9) | 0 (0.0) |
| ESHAP | 0 (0.0) | 128 (14.8) | 19 (5.4) | 8 (7.1) | 2 (4.9) |
| ESHAP followed by HSCT | 0 (0.0) | 11 (1.3) | 2 (0.6) | 0 (0.0) | 0 (0.0) |
| MINE | 1 (0.1) | 49 (5.7) | 39 (11.1) | 16 (14.2) | 6 (14.6) |
| MINE followed by HSCT | 0 (0.0) | 0 (0.0) | 11 (3.1) | 0 (0.0) | 1 (2.4) |
| Miscellaneous regimen | 10 (1.2) | 104 (12.0) | 81 (22.9) | 42 (37.2) | 19 (45.2) |
| Miscellaneous regimen followed by HSCT | 1 (0.1) | 15 (1.7) | 19 (5.4) | 1 (0.9) | 0 (0.0) |
| ^a)^ CHOP-like treatments included cyclophosphamide, vincristine, doxorubicin, or prednisolone regimens (minimum two of these options).  R-CHOP, rituximab, cyclophosphamide, vincristine, doxorubicin, prednisolone; CHOP, cyclophosphamide, vincristine, doxorubicin, prednisolone; ICE, ifosfamide, carboplatin, etoposide; DHAP, dexamethasone, cytarabine, cisplatin; ESHAP, etoposide, methylprednisolone, cytarabine, cisplatin; MINE, mesna, ifosfamide, mitoxantrone, etoposide; HSCT, hematopoietic stem cell transplantation | | | | | |

CHOP, cyclophosphamide, hydroxydaunorubicin, oncovin, and prednisone; DHAP, dexamethasone, cytarabine, and cisplatin; ESHAP, etoposide, methylprednisolone, cytarabine, and cisplatin; HSCT, hematopoietic stem cell transplantation; ICE, ifosfamide, carboplatin, and etoposide; MINE, mesna, ifosfamide, mitoxantrone, and etoposide; R-CHOP, rituximab-CHOP

Supplementary Table S2. Economic burdens of patients with second-line failure

|  | **Second-line non-failure**  **(N = 220)** | **Second-line failure ^a)^**  **(N = 644)** |
| --- | --- | --- |
| **Total medical cost, median (IQR)** | $34,182 (20,000–51,807) | $42,706  (22,608–66,769) |
| Total inpatient cost | $22,833  (11,697–36,013) | $34,370  (18,332–60,206) |
| Total medical cost related to HSCT | $19,492 (15,742–27,306) | $24,121 (16,343–38,441) |
| **Medical costs for each treatment line, median (IQR)** | | |
| First-line treatment ^b)^ | $33,113  (26,288–43,570) | $32,149  (25,633–41,299) |
| Second-line treatment ^c)^ | $28,196  (15,619–45,712) | $19,185  (10,373–34,240) |

^a)^ Second-line failure included progression to third-line treatment or death during second-line treatment.
^b)^ From diagnosis to second-line treatment
^c)^ From second-line to third-line treatment, death, or the end of the study period;
IQR, interquartile range; HSCT, hematopoietic stem cell transplantation; 1 USD = 1086.30 KRW (2020 exchange rate)

**Supplementary Table S3. Cumulative mean disease-related cost based on** **third-line treatment**

| **Time from second-line treatment (months)** | **Patients who received  third-line treatment (N = 353)** | **Patients who did not receive  third-line treatment^*^ (N = 511)** |
| --- | --- | --- |
| 1 | 32,533.11 | 37,381.51 |
| 2 | 38,089.23 | 43,500.34 |
| 3 | 44,250.01 | 48,471.08 |
| 4 | 50,879.88 | 52,703.34 |
| 5 | 57,042.43 | 56,027.19 |
| 6 | 61,698.50 | 58,295.24 |
| 7 | 65,968.23 | 60,307.76 |
| 8 | 70,213.92 | 61,505.49 |
| 9 | 74,006.87 | 62,288.10 |
| 10 | 76,682.36 | 62,892.93 |
| 11 | 78,653.42 | 62,892.93 |
| 12 | 80,417.65 | 62,951.52 |
| 13 | 81,748.42 | 63,415.88 |
| 14 | 83,081.29 | 63,783.92 |
| 15 | 84,027.25 | 63,982.18 |
| 16 | 84,939.15 | 64,145.30 |
| 17 | 85,795.75 | 64,422.42 |
| 18 | 86,343.31 | 64,643.09 |
| 19 | 87,131.18 | 64,784.45 |
| 20 | 87,758.62 | 64,979.31 |
| 21 | 88,450.92 | 65,139.45 |
| 22 | 89,224.45 | 65,251.36 |
| 23 | 89,970.98 | 65,391.68 |
| 24 | 90,458.54 | 65,521.46 |
| 25 | 90,869.00 | 65,645.09 |
| 26 | 91,272.27 | 65,760.18 |
| 27 | 91,540.84 | 65,868.72 |
| 28 | 91,757.07 | 65,960.09 |
| 29 | 92,096.06 | 66,072.31 |
| 30 | 92,368.73 | 66,185.22 |
| 31 | 92,383.67 | 66,296.27 |
| 32 | 92,620.66 | 66,401.00 |
| 33 | 92,961.30 | 66,501.23 |
| 34 | 93,226.97 | 66,623.69 |
| 35 | 93,344.10 | 66,691.37 |
| 36 | 93,429.00 | 66,835.36 |

* Patients who did not receive third-line treatment included those who died during the second-line treatment.


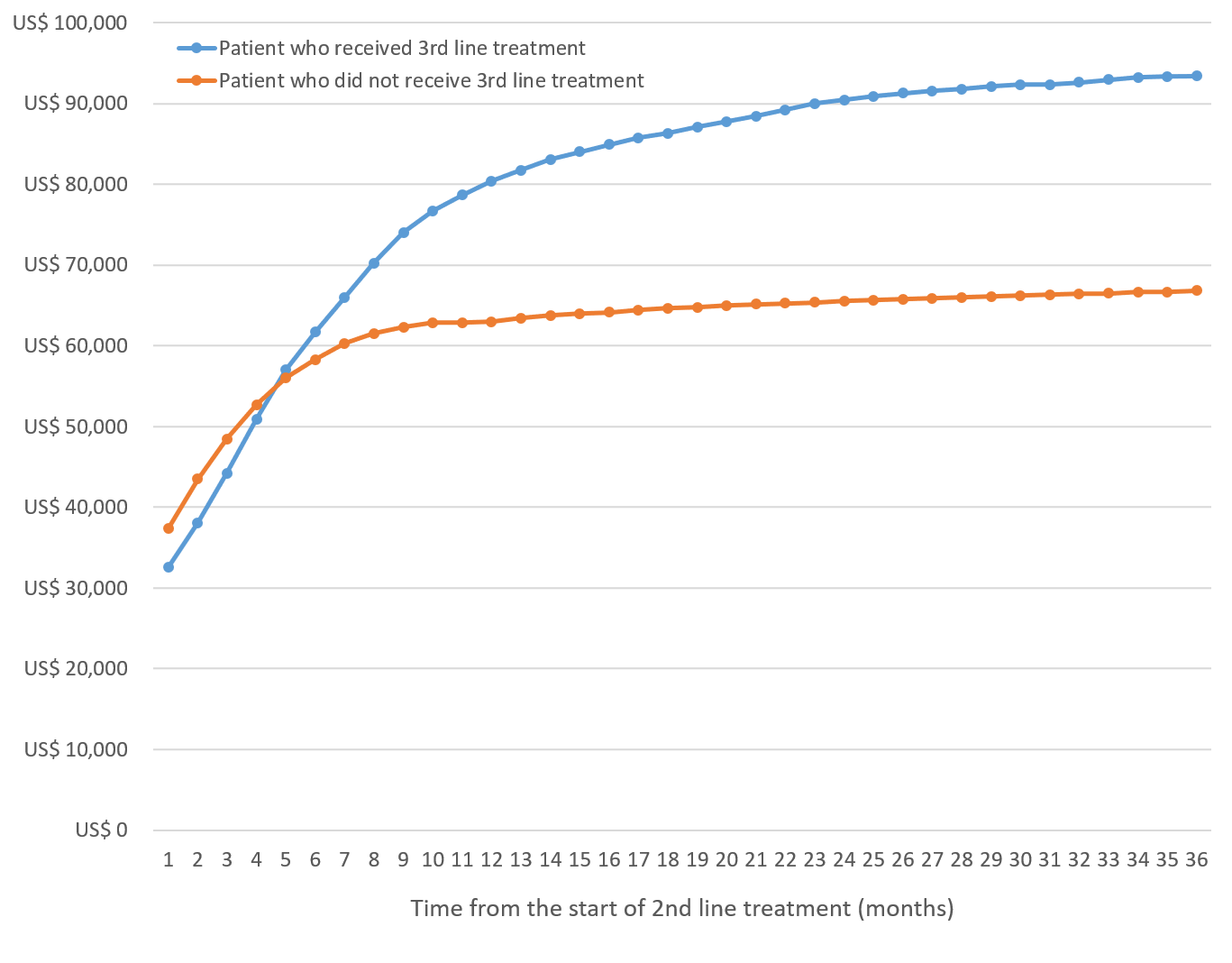


Supplementary Figure S1. Cumulative mean disease-related costs based on third-line treatment
* Patients who did not receive third-line treatment included those who died during second-line treatment.
